# A Micrometer Object-Finder

**Published:** 1857-01

**Authors:** R. J. Farrants


### ARTICLE XI.

*A Micrometer Object-Finder.* By R. J. Farrants, Esq. Read April 30, 1856.

A sure and ready method of determining the position of a microscopic object on the glass slide which contains it, so that it may be registered, and the object be brought into the field of view of the microscope, at any time and with any instrument, is still a desideratum with microscopic observers. Ingenious suggestions have indeed been offered from time to time, with a view to supply this want; but that none of them have fulfilled the requirements may be inferred from the fact that no one of them has obtained general acceptance. I do not doubt that an object may be found, if its place on the glass slide has been accurately registered, with any of the "Finders" at present before the public; still, though the result may be sure, with none of them is the method of determining the position of the object, in the first instance, of ready application.

The plan of Mr. E. G. Wright, of Hereford, published in the "Quart. Journ. of Micr. Science," vol. 1, p. 301, consists in fixing to or engraving upon the stage of the microscope two scales at right angles to each other, and reading off from these scales the numbers against the end of the glass slip, and the lower edge of the object-plate, when the object is in the field. This plan may be efficient, but is of very limited applicability, being useful, even to the person who adopts it, only with the particular instrument with which the position of the object has been registered.

Other methods more generally applicable have not yet been very generally applied. It is, therefore, not unreasonable to presume that all such are deficient in some quality essential to their utility. It appears to me that the deficiency is their not affording ready means of determining the position of an object in the first instance. With most of them it is necessary that the "finder" should be applied to the stage, and be accurately centered before any use can be made of its scales. This is the case with the "Universal Indicator" of Mr. J. W. Bailey, described in the "Quart. Journ. of Micr. Science," vol. iv, p. 55, and with the "Finder" of Mr. T. E. Amyot, described in the same work, vol. iv, p. 151. On the plan of Mr. E. G. Wright, also, it is necessary to "adjust the movable stage exactly square," before the scales are of any use. Now the necessity of observing these conditions as preliminaries to the use of the finder, must frequently prevent its being employed, and that too in cases where the want of it is most felt. It must often happen that an observer in examining a glass slide meets with some remarkable object, a frustule or valve of *Diatomaceæ*, for instance, a peculiar crystal, or some particular cell occurring either in vegetable or animal structure, which he would like to examine more attentively at another time; but not expecting, and, therefore, not having provided for his having occasion to register the position of any object, he is not able to do so, or at least, as the first step, before he can adjust the stage and apply the scales, he must lose the object whose position he desires to register, chiefly because of the difficulty of finding it; then, after the necessary adjustments have been made, the object must again be sought for. In practice, no doubt, the observer frequently concludes that this tedious operation may as well be deferred until he shall want the object for further examination, and so the registry is not made at all. In using the finder of Mr. Tyrrell, (see "Quart. Journ. of Mic. Science," vol. 1, p. 234,) it is true, that there are no preliminary adjustments to be made, yet with it also, the object must be lost in removing the glass from the stage to place it in the finder, and must be again found before its position can be registered.

The terms latitude and longitude have been already made use of, in reference to the place of a microscopical object on a glass slide. The convenience and aptness of the terms are a sufficient reason for continuing to employ them.

Latitude refers to the position of an object, with respect to the width of the glass. Longitude refers to its position with reference to the length of the glass. Latitude, therefore, will be determined by a line parallel to the sides of the slide which would pass through the object, and the distance of such line from one or other of the sides of the slide, expressed in terms of a scale that has been determined upon, will denote the latitude of the object. The distance from one of the ends of the slip of a line parallel thereto, expressed in like manner, will be the longitude. Latitude, then, will be measured by horizontal lines, and longitude by vertical ones.

Now, if the latitude of an object can be determined with precision, and if the line corresponding thereto, can with accuracy be made to coincide with the transverse diameter of the field of view, the object itself may be brought into view by simply moving the glass slide along the stage.

The ledge at the bottom of the object-plate, against which the slide rests when the body of the microscope is in an inclined position, is a line from which the latitude may be conveniently estimated, for as the lower edge of the slide rests against this ledge, the distance from it to the line on which the object is situate, is the latitude required. For determining the longitude, it is convenient that the object-plate be furnished with a stop on one side, (preferably the left,) against which the glass slide may be made to abut. This may consist of a narrow slip of brass, half an inch in length, projecting a little above the surface of the plate, and having two pins on its under surface, to drop into two small holes drilled in the object-plate to receive them. Such a stop is applied and removed in an instant, and affords a line from which the longitude may be measured, as certainly and as conveniently as latitude is measured from the ledge on which the slide rests.

The scales to be used in determining the position of an object, are ruled on a slip of glass, or other material, of the size ordinarily used for microscopic purposes, viz. 3 inches by 1 inch. Let the centre inch be ruled with horizontal and vertical lines, at regular distances apart, say 50 to the inch; the superficial square inch will thus be covered with small squares: this done, the observer is supplied with the means of accurately determining the position of any object. These scales are available at any moment, no preliminary arrangements are necessary before they can be used, and the readings being made under the microscope, may be made with greater exactness than is possible in reading scales applied to the stage, which can be seen only with the eye, at most aided by a lens.

I have had ample experience of the efficiency and utility of the method of registration, the details of which are given below, having used it for several years, though with very imperfect scales; indeed, they were nothing more than lines 25 to the inch, scratched with a writing diamond on the centre inch of an ordinary glass slip, the vertical and horizontal lines being on different glasses, or on different sides of the same glass. These scales afforded the means of registering and finding any object with the $\frac{2}{3}$-inch object-glass, but the divisions were neither sufficiently regular or minute enough to be used with advantage with an object-glass of higher power. Of late, through the kindness of Mr. G. Jackson, (to whom I am much indebted for the trouble he has taken in the matter, and for which I pray him to accept my best thanks,) I have been enabled to make trial of different scales, ruled with that precision and neatness which are so well known to and appreciated by all who make use of the micrometers ruled by that gentleman. These scales, and the mode of using them, I will describe as briefly and intelligibly as I can.

Since objects are usually placed as nearly as possible in the centre of the slide on which they are mounted, it rarely happens that any specimen is found within an $\frac{1}{8}$th of an inch of either edge of the slide, and the $\frac{3}{4}$ths of an inch square in the middle of the slide is the space within which most objects will be found; and since nothing is gained by having more lines ruled than are actually wanted, while the counting is thereby rendered more troublesome, it has been thought better not to extend the ruling over a greater surface than $\frac{3}{4}$ths of an inch square.

The scales ruled by Mr. Jackson, which I now use, and would recommend, are formed by horizontal and vertical lines, ruled on the same surface of the glass, at a distance of $\frac{1}{100}$th of an inch apart; every fifth line is thicker, and consequently more conspicuous than the rest, besides, the centres of both series are distinguished by double lines; and by these means the counting is greatly facilitated: there are in each series 72 lines, (the centres being double,) which include 70 spaces, of which 35 lie on each side of the central double line in each direction; a space $\frac{7}{10}$ (.7) of an inch square in the middle of a glass slip is thus covered with a series of squares, 4900 in number, the sides of which are $\frac{1}{100}$th of an inch linear, and the area $\frac{1}{10000}$th of a square inch; of these, the field of view with $\frac{1}{4}$-inch object-glass, will include nearly 4; or if any square be brought to the centre of the field, there will also be visible the half of 4 squares, one on each side of the central one, and nearly a quarter of 4 others, one at each corner of that which occupies the centre of the field.

The mode of using these scales is as follows: If, for example, it be wished to register the latitude of an object in the field of the microscope, (the lower edge of the glass slide, it is presumed, resting against the ledge of the object-plate,) bring the object as nearly as possible to the centre of the field; then remove the slide from the stage, and put in its place the ruled slip; observe which line coincides with the transverse diameter of the field, that is, with the position of the object, then count (upwards, as it appears in the microscope) from this line to the end of the series: suppose the 45th line is that which corresponds with the position of the object, then 45 will denote the latitude of that object, that is, its distance from the lower edge of the slide. At any future time, and with any instrument, this object can be readily found; to effect this, place the ruled slip on the object-plate, the lower edge resting evenly against the ledge, then bring the upper line (as it appears) of the horizontal series into view, and count 45 from it, (this is easily and quickly done by taking advantage of the conspicuousness of every fifth line,) make that line coincide with the transverse diameter of the field of view; the ruled slip may then be removed from the stage, and the slide containing the object be put in its place; the object sought now lies somewhere on the line which corresponds with the transverse diameter of the field, and if the glass be slowly moved across the stage will soon come into view.

It is generally sufficient to determine with exactness the latitude of an object: if, however, it be thought desirable to register the longitude also, this may be done by proceeding in a manner altogether analogous to that just described, the only difference being that it is the position of a vertical line passing through the object that is now to be ascertained, and its distance from the end of the slide. Now it is that the lateral stop will be found convenient; it is not, however, indispensable, since a temporary expedient may be made to supply its place, or the lateral edge of the object-plate may be taken as the line from which the longitude is measured; in that case, the end of the slide containing the object must be brought even with the edge of the object-plate, and the object being brought into view by means of the lateral stage movements, the slide is then to be removed, and the ruled slip put in the same position, that is, with its end abutting against the lateral stop, or exactly even with the side of the object-plate, as the case may be; the vertical line coinciding with the vertical diameter of the field of view will then correspond with a vertical line on which the object is situate; suppose this line is the 40th (from the right of the series, as seen in the microscope,) the number 40 will then express the longitude, and will denote its distance from the left end of the slide. It is scarcely necessary to remark, that the slide, as well as the ruled slip, must always be placed on the stage in the same way, that is, with the same edge always uppermost: to ensure this it is well to adhere constantly to some plan; for instance, the rule may be always to keep the label on the slide to the right hand.

If the longitude has been registered as well as the latitude, the object will be found by proceeding as follows: First find the latitude by bringing the horizontal line on the ruled slip denoted by the number into the centre of the field; then, taking care that the end of the slip is close up to the lateral stop, or even with the edge of the object-plate, bring into the centre of the field the vertical line denoted by the register; the ruled slip may then be removed from the stage, and the slide with the object being put in its place, the object will be in the field; and if the observations and manipulation have been made with correctness, will be at or near the centre.

If the eye-piece have an indicator it will be convenient to bring it into the field, and to move the object to its point; the line on the ruled slip which corresponds with it may then be ascertained with the greatest exactness. If there be not an indicator, still, as the eye-glasses will seldom be so clear as not to have some mark or speck of dust, any such accidental mark that may be observed may be made to serve the same purpose as the point of the indicator.

An object once found need never be lost till its position has been determined and registered; for without moving the stage or object-plate, the slide with the object may be taken off the stage, and the ruled slip be put in its place: this may be moved upwards by the hands while the horizontal lines are being counted, and to one side, (the right,) to enable the observer to count the vertical ones; these being duly noted, the ruled slip may be taken away, and the glass with the object be again put on the stage. If no movement of the stage have been made the object should be seen in the same position as at first; the accuracy of the register can thus be at once ascertained, or any error be detected and corrected.

As may be expected, it in fact seldom happens that any line on the ruled slip will exactly coincide with the position of an object, which is more frequently found to lie in the interval between two lines. It is better, therefore, to make the register refer to the spaces instead of to the lines. The number 45 then, for example, denoting the latitude of an object, will indicate that its position is in the space expressed by that number.

To register the position of an object, it is only necessary to put down in figures the numbers which denote the spaces observed; for instance, the position of the object just supposed to have been ascertained would be registered thus:

Lat. 45. Long. 40.

It will save trouble, if an arbitrary significance be given to position. Thus it may be determined that the first number shall always refer to latitude, in which case the register might stand thus:

$$\frac{45}{40} \text{ or } 45:40.$$

I prefer the latter, because the figures better admit of any appendix which may be used to modify their significance. For example, the object whose position is found to correspond with the square denoted by 45 : 40, may lie nearer to the upper line than to the lower one, or the contrary; now this may be clearly indicated by a short line added to the figures. Thus, if the object lie nearer the upper line, that may be indicated by a short line above the figures, $\overline{45}$; if the object lie nearer the lower line, the fact may be indicated by a line below the figures thus, $\underline{45}$. Similarly the greater proximity of an object to the vertical line on the right or left of it may be shown by a short line before or after the figures, as |40 40|, the meaning of the line so placed is obvious. Again, if the position of the object coincide exactly with a line on the ruled slip, that may be denoted by drawing a line through the figures thus, ~~45~~; if then the position of any object be registered thus, ~~45~~ : |40, it will signify, that its latitude corresponds with the 45th horizontal line of the ruled slip, while its longitude corresponds with the 40th vertical space, but that it is nearer to the line on the left side. In this way then, four positions, in respect of each square, may be clearly pointed out, and the area of each square being the $\frac{1}{10000}$th of a square inch, it follows that the place of an object can be assigned, with an exactness nearly approaching the $\frac{1}{40000}$th of a square inch. As the field of view with the $\frac{1}{4}$-inch object-glass with the first eye-piece shows nearly the whole of four squares, or the whole of one square, and a part greater or less of eight others, it is scarcely possible, with only moderate care, that an observation should be so inaccurate as that an object whose position has been registered, should not be brought into the field of that glass. If sufficient attention be paid in ascertaining and recording the position of the object, it may almost as surely be placed in the field of an $\frac{1}{8}$th or a $\frac{1}{12}$th.

In registering the position of an object, as determined by these scales, a somewhat different plan from that already described, may be and is adopted by some. Thus, taking advantage of the clear determination of the centres of both scales, with a view to reduce the counting to a minimum, the reckoning may proceed from the centre in all directions. The horizontal lines may be counted from the centre upwards and downwards for latitude; the vertical ones too, may be counted from the centre, right and left for longitude. On this plan, the latitude of an object, registered on the previous plan as 45, would be registered 10 below the centre. If it were 25 on the first plan, according to this one, it would be 10 above the central line; this line being taken to represent the equator, the terms north and south, may be used with reference to it. In like manner, east and west may be used in reference to longitude, to signify the position of a vertical line right or left of the central one.

The manner of registering the observations will make no difference in the result, and is a mere matter of detail which every individual may settle for himself. The only important things, are that both the observation and registration be accurate.—*Quarterly Jour. Micros. Sci.*

